# Gastrointestinal Involvement in a Patient with Chronic Lymphocytic Leukemia

**DOI:** 10.4274/balkanmedj.galenos.2019.2019.9.51

**Published:** 2019-12-20

**Authors:** Devrim Çabuk, Fatih Ballı, Yasin Yılmaz, Ali Erkan Duman, Kazım Uygun

**Affiliations:** 1 Department of Internal Medicine, Kocaeli University School of Medicine, Division of Medical Oncology, Kocaeli, Turkey; 2 Department of Internal Medicine, Kocaeli University School of Medicine, Kocaeli, Turkey; 3 Department of Pediatrics, İstanbul University İstanbul School of Medicine, İstanbul, Turkey; 4 Department of Internal Medicine, Kocaeli University School of Medicine, Division of Gastroenterology, Kocaeli, Turkey

A 70-year-old man was admitted to the hospital with symptoms of fatigue and dyspnea. A total blood count showed leukocytosis with an increased lymphocyte count. In a short time, his white blood cell (WBC) count had increased to 200 000 (WBC: 217.00×10^9^/L, lym: 175.00×10^9^/L, and neu: 22.30×10^9^/L) and flow cytometry revealed a phenotype that was positive for CD20, CD19, and CD5 and negative for FMC7. CD 23 was highly positive (70.09%), and there was co-expression of CD5 and CD19 (68.46%). Surface membrane immunoglobulin levels were low (5.27%). This phenotype was typical of a diagnosis of chronic lymphocytic leukemia (CLL).

Abdominal and thorax computerized tomography (CT) revealed hepatosplenomegaly, peritoneal carcinomatosis, para-aortic and celiac lymphadenopathies, and diffuse wall thickening in the rectum. The patient was referred to the medical oncology department with a diagnosis of rectal cancer and peritoneal metastasis. A CT of the thorax showed bilateral pleural effusion and nodular lesions in the bilateral lung parenchyma.

Gastroscopy revealed that the gastric mucosa was hyperemic; ulcerative lesions on bilateral sides of the corpus, and multiple pearlescent lesions in the post bulbar region were detected (Figure 1). A colonoscopy showed advanced inflammation of the rectal mucosa. The mucosal surface was irregular and occasionally ulcerative. Gastroscopic biopsies revealed lymphocytic infiltration in the post bulbar region of the duodenum, and greater curvature of the corpus and colonoscopic biopsies showed lymphocytic infiltration in the descending part of colon and rectum. Microscopically dense mucosal and submucosal lymphocytic infiltrations were detected. Immunohistochemistry was positive for CD20, CD5, and CD23 (Figure 2). The test for cyclin D1 was negative. These findings indicated the infiltration of CLL cells into the gastrointestinal system.

During the course of the disease, the patient had renal insufficiency, neutropenia, and fever. The patient was thought to be immunocompromised due to the disease (CLL) or possibly due to bone marrow suppression as a result of sepsis. Hydration and broad spectrum antibiotics were started. The patient had increased symptoms of dyspnea and hypoxia. The dyspnea and hypoxia were thought to be associated with lymphocyte infiltration in the lungs and pneumonia. Despite treatment, the patient died because of respiratory failure and sepsis.

Gastrointestinal involvement (GI) is an important and rare complication of CLL. This complication can be due to lymphocytic infiltration or concomitantly occurring colon carcinoma (1,2,3). GI generally occurs only when Richter syndrome develops, which is the transformation of CLL to diffuse, large B-cell lymphoma.

Gastrointestinal manifestations are sometimes asymptomatic in CLL patients. GI can caused by esophageal varices due to portal hypertension, protein-losing enteropathy, and colitis (4,5). A prompt and detailed endoscopic examination is essential for these patients.

In conclusion, gastrointestinal evaluation of patients with CLL should be performed because these patients are at increased risk of additional malignancy. However, endoscopy with biopsy might reveal GI involvement rather than a secondary malignancy. This should be kept in mind and treatment should be initiated as soon as possible.

## Figures and Tables

**Figure 1:**
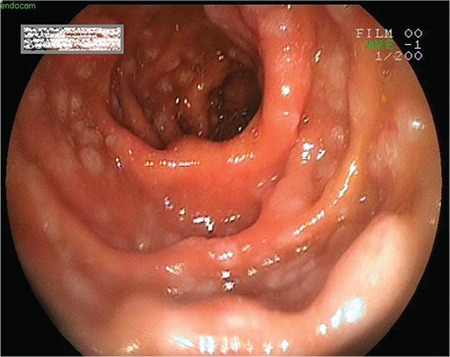
Gastroscopy showed multiple pearlescent lesions on post bulbar region.

**Figure 2:**
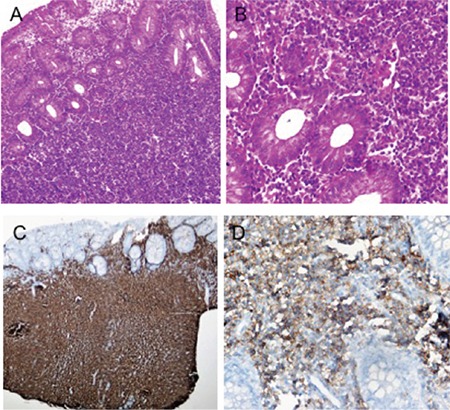
Biopsy specimens of the colonic mucosa. A) Extensive lymphoid infiltration that destroyed gland structures (H&E ×100). B) Diffuse mucosal infiltration of lymphocytes with dense clumped chromatin and scanty cytoplasm (H&E ×400). C) Diffuse staining with CD20 (×40). D) CD5 positivity of infiltrating lymphocytes (×400).

## References

[1] Arkkila PE, Nuutinen H, Ebeling F, Elonen E, Kärkkäinen P, Karjalainen-Lindsberg ML (2008). Colonic involvement in a patient with chronic lymphocytic leukaemia. Gastroenterol Res Pract.

[2] Kuse R, Lueb H (1997). Gastrointestinal involvement in patients with chronic lymphocytic leukemia. Leukemia.

[3] Kyo K, Sameshima S, Tanaka Y, Murayama K, Shimano S, Kojima M, et al (2004). Rectal cancer associated with chronic lymphocytic leukemia. J Gastroenterol.

[4] Wilputte JY, Martinet JP, Nguyen P, Damoiseaux P, Rahier J, Geubel A (2003). Chronic lymphocytic leukemia with portal hypertension and without liver involvement: a case report underlining the roles of increased spleno-portal blood flow and "protective" sinusoidal vasoconstriction. Acta Gastroenterol Belg.

[5] Scharschmidt BF (1978). Chronic lymphocytic leukemia presenting as colitis. Digest Dis Sci.

